# Serotype-conversion in *Shigella flexneri:* identification of a novel bacteriophage, Sf101, from a serotype 7a strain

**DOI:** 10.1186/1471-2164-15-742

**Published:** 2014-08-30

**Authors:** Richa Jakhetia, Aruna Marri, Jonas Ståhle, Göran Widmalm, Naresh K Verma

**Affiliations:** Division of Biomedical Science and Biochemistry, Research School of Biology, The Australian National University, Bldg. 134 Linnaeus Way, Canberra, ACT 0200 Australia; Department of Organic Chemistry, Arrhenius Laboratory, Stockholm University, S-106 91 Stockholm, Sweden

**Keywords:** *Shigella flexneri*, Bacteriophage, O-antigen modification, Serotype-conversion

## Abstract

**Background:**

*Shigella flexneri* is the major cause of bacillary dysentery in the developing countries. The lipopolysaccharide (LPS) O-antigen of *S. flexneri* plays an important role in its pathogenesis and also divides *S. flexneri* into 19 serotypes. All the serotypes with an exception for serotype 6 share a common O-antigen backbone comprising of N*-*acetylglucosamine and three rhamnose residues. Different serotypes result from modification of the basic backbone conferred by phage-encoded glucosyltransferase and/or acetyltransferase genes, or plasmid-encoded phosphoethanolamine transferase. Recently, a new site for O-acetylation at positions 3 and 4 of Rha^III^, in serotypes 1a, 1b, 2a, 5a and Y was shown to be mediated by the *oacB* gene. Additionally, this gene was shown to be carried by a transposon-like structure inserted upstream of the *adrA* region on the chromosome.

**Results:**

In this study, a novel bacteriophage Sf101, encoding the *oacB* gene was isolated and characterised from a serotype 7a strain. The complete sequence of its 38,742 bp genome encoding 66 open reading frames (*orfs*) was determined. Comparative analysis revealed that phage Sf101 has a mosaic genome, and most of its proteins were >90% identical to the proteins from 12 previously characterised lambdoid phages. In addition, the organisation of Sf101 genes was found to be highly similar to bacteriophage Sf6. Analysis of the Sf101 OacB identified two amino acid substitutions in the protein; however, results obtained by NMR spectroscopy confirmed that Sf101-OacB was functional. Inspection of the chromosomal integration site of Sf101 phage revealed that this phage integrates in the *sbcB* locus, thus unveiling a new site for integration of serotype-converting phages of *S. flexneri*, and determining an alternative location of *oacB* gene in the chromosome. Furthermore, this study identified *oacB* gene in several serotype 7a isolates from various regions providing evidence of O-acetyl modification in serotype 7a.

**Conclusions:**

This is the first report on the isolation of bacteriophage Sf101 which contains the *S. flexneri* O-antigen modification gene *oacB*. Sf101 has a highly mosaic genome and was found to integrate in the *sbcB* locus. These findings contribute an advance in our current knowledge of serotype converting phages of *S. flexneri*.

**Electronic supplementary material:**

The online version of this article (doi:10.1186/1471-2164-15-742) contains supplementary material, which is available to authorized users.

## Background

The burden of bacterial dysentery due to shigellosis continues to be a major concern affecting more than 165 million people annually. *S. flexneri* is the primary cause of endemic shigellosis prevalent in the developing countries, and is the most frequently isolated species world-wide [1].

In *S. flexneri*, the O-antigen structure of the LPS, is one of the key virulence determinants required for its pathogenesis and protection against the host defence [2]. The O-antigen of *S. flexneri*, with the exception of serotype 6, consists of a polysaccharide backbone comprised of the repeating tetrasaccharide units (N*-*acetylglucosamine and three rhamnose residues), and modifications to the basic O-antigen backbone by the addition of glucosyl, O-acetyl, or phosphoethanolamine groups to one or more sugars, give rise to various different serotypes of *S. flexneri* [3–6]. To date at least 19 serotypes of *S. flexneri* have been identified [7].

The genes responsible for glucosylation of *S. flexneri* O-antigen, *gtrA*, *gtrB* and *gtr*(type), are arranged in a single operon known as the *gtr* cluster [8–13]. Whereas, the acetylation is mediated by a single gene ‘*oac’* encoding O*-*acetyltransferase [14]. While the glucosylation is known to occur on any of the sugar residues, *oac-*encoded O*-*acetylation occurs at position 2 of Rha^I^ residue. Additionally, the glucosyl or the O*-*acetyl modification genes are carried by bacteriophages, which mediate serotype-conversion by integrating into the host chromosome [3]. Six such serotype converting phages have been identified: SfI, SfII, SfIV, SfV, and SfX, which encode the *gtr* gene cluster, and Sf6 which encodes the *oac* gene [10, 15–18]. In contrast to the phage-encoded glucosylation and O-acetylation, the phosphoethanolamine modification at position 3 of Rha^II^ and/or Rha^III^, is encoded by a plasmid-borne *opt* gene [6].

Recently, new sites for the O-acetylation of *S. flexneri* O-antigen have been identified. These are O-acetylation at position 3 (major) and 4 (minor) of Rha^III^ (3/4-O-acetylation) in serotypes 1a, 1b, 2a, 5a, Y, 6 and 6a, and at position 6 of GlcNAc in serotype 2a, 3a, and Y [19–22]. Moreover, the degree of 3/4-O-acetylation has also been shown to vary between the ranges 30-70% (at position 3) and 15-30% (at position 4), within the strains of one serotype [21]. Another recent study revealed that the 3/4-O-acetylation modification in *S. flexneri* is mediated by the *oacB* gene, which was shown to be carried by a transposon-like structure located upstream of the *adrA* gene on the chromosome [23]. In this study, we report the isolation and characterisation of a novel bacteriophage Sf101, from a wild type serotype 7a strain, and show for the first time that *oacB* gene was located on the intact genome of the prophage, Sf101. The complete genome sequence of the Sf101 phage was determined. Comparative genomics used for highlighting important genetic similarities with other lambdoid phages indicated that the Sf101 phage has a mosaic genome and Sf6-like genome architecture. Analysis of a specific chromosomal integration site for phage Sf101 revealed that the phage integrates into the *sbcB* locus, thus identifying a new site for the integration of the serotype converting phages of *S. flexneri*, and a new location of *oacB* in *S. flexneri* chromosome. Additionally, this study also identified *oacB* gene in several serotype 7a isolates from various geographical regions, suggesting that the 3/4-O-acetylation modification is not uncommon in serotype 7a of *S. flexneri*.

## Methods

### Bacterial strains, bacteriophage and media

The induction of bacteriophage Sf101 from serotype 7a *S. flexneri* strain SFL1683 was performed using UV irradiation protocol described by Adam et al. [8]. Bacteriophage stocks were prepared by picking a single plaque, propagating on serotype Y strain (SFL124) [24], and precipitating phage using polyethylene glycol, as described in Sambrook et al. [25]. *S. flexneri* strain SFL1691 (serotype 7a) lacking the 3/4-O-acetylation on the Rha^III^ was used as host for Sf101-OacB functional analysis. *E. coli* JM109 was used for cloning experiments.

Bacteria were grown in Luria–Bertani (LB) broth or LB agar supplemented with Chloramphenicol (25 μg/ml) or Erythromycin (250 μg/ml) when appropriate. NZCYM broth was employed for routine propagation of phage.

### DNA methods

Sf101 phage DNA was isolated from purified phage stock by treatment with Proteinase K as described by Sambrook et al. [24]. Bacterial genomic DNA was isolated using GE Healthcare genomic DNA isolation kit (GE Healthcare), according to the manufacturer’s instructions. Plasmid isolation was performed using the Axyprep Plasmid Miniprep kit (Axygen Biosciences). Primers used in this study were synthesized by Sigma-Aldrich and are listed in Additional file 1: Table S1. PCR amplification was performed using the PfuUltra II Fusion HS DNA Polymerase (Stratagene) according to the manufacturer’s directions. Purification of the PCR products was achieved by using the Wizard SV Gel and PCR Clean Up System (Promega). DNA sequencing was performed using Big Dye Terminator v3.1 Cycle Sequencing kit as recommended by the manufacturer and were run on an AB 3730 capillary sequencer at Biomedical Resources Facility, John Curtin School of Medical Research, Australian National University.

Sf101 *oacB* was amplified using Sf101 phage DNA as template, using primers Sf101-OacB-Fwd and Rev. The purified product was then cloned into vector pBC SK + (Stratagene) to generate plasmid pNV2073. Erythromycin gene, PCR amplified from plasmid pTRKH2 using Em primer pair was then cloned into pNV2073 to generate plasmid pNV2074. The recombinant plasmids were transformed by electroporation and maintained in JM109 cells. pNV2074 was also introduced into *S. flexneri* strain SFL1691 to produce SFL2516.

### Electron microscopy

The purified phage was absorbed on carbon-coated copper grids, negatively stained with 2% phosphotungstic acid (pH 7.0) and visualized with a Hitachi H7000 transmission electron microscope.

### Host range determination

The host range of phage Sf101 was determined as described previously [17]. Briefly, different dilutions of phage stock were made in SM buffer; 5-10 μl of each dilution was then spotted on required bacterial lawn on an agar plate. Lytic activity (clear zone encompassing the phage drop) was examined following overnight incubation at 37°C.

### Sequencing of phage genomic DNA

The purified Sf101 phage DNA was sequenced using the Ion Torrent PGM 314 chip (Life Technologies) at the Australian Genome Resource Facility, University of Queensland. The reads generated were assembled into contigs using CLC Genomics Workbench (Ver 5.5.1, CLC Bio). To close the gaps between the contigs and re-sequence the regions of low-quality, the desired segments were PCR amplified using phage DNA as template and the purified PCR products were sequenced as described above.

### Analysis of sequence

Open reading frames were determined using CLC Main Workbench (Ver 5.5.1, CLC Bio) and NCBI ORF finder program. Genes within *orfs* were predicted based on homologies to the known genes found by BlastN and BlastP searches and the presence of Shine-Dalgarno ribosome binding sites. The Rho-independent terminators were identified using ARLOND terminator finding program [26] and tRNAscan-SE search server was used to scan for tRNA [27].

The protein level alignments were performed using ClustalW [28] and BioEdit Sequence Alignment Editor [29]. The accession numbers for the phages used for comparative genomics were: CUS-3 (CP000711), Sf6 (NC_005344), HK544 (NC_019767), mEP043 c-1 (NC_019706), SfX (NC_017328), mEp213 (NC_019720), mEp234 (NC_019715), P22 (NC_002371), epsilon34 (NC_011976), Ime10 (NC_019501), HK446 (NC_019714), Phage 2851 (FM180578).

### Preparation of O-polysaccharide and NMR spectroscopy

Lipopolysaccharide of strains SFL1683, SFL1691 and SFL2516 were isolated by the phenol water extraction of dried bacterial cells [30]. Lipid-free polysaccharide (PS) was prepared by treatment of the LPS with dilute acetic acid followed by purification using gel permeation chromatography [31].

^1^H NMR spectra of the LPS in D_2_O solution were recorded at 80°C on a Bruker Avance II 500 MHz spectrometer. 1D and 2D NMR experiments [32] of the PS were recorded in D_2_O solution on Bruker Avance 500 MHz and Bruker Avance III 700 MHz spectrometers, equipped with 5 mm TCI Z-Gradient CryoProbes, at 24°C for strain SFL1683 and at 20°C for strain SFL2516. Data processing was performed using vendor-supplied software. Chemical shifts are reported in ppm using internal sodium 3-trimethylsilyl-(2,2,3,3-^2^H_4_)-propanoate (TSP, δ_H_ 0.00) or external 1,4-dioxane in D_2_O (δ_C_ 67.40) as references.

### Availability of supporting data

The nucleotide sequence of Sf101 phage reported in this article has been deposited in the GenBank database as accession number KJ832078.

## Results and discussion

### Isolation of Sf101

A novel bacteriophage named Sf101 was induced and isolated from serotype 7a, strain SFL1683. Phage Sf101 formed clear, round plaques when plated onto the indicator strain SFL124 (serotype Y). In order to confirm that SFL1683 is the host strain of Sf101 phage, Southern hybridization of *HindIII* digested genomic DNA of the host, using DIG-labelled Sf101 as a probe was performed. Results confirmed the presence of phage genome integrated into the host chromosome (data not shown).

### Morphology of Sf101

**Figure 1:**
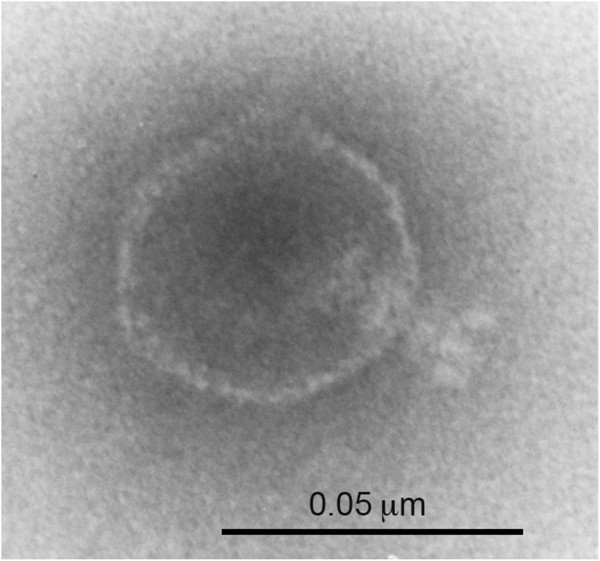
**Electron micrograph of phage Sf101 negatively stained with 2% phosphotungstic acid.** Scale bar, 50 nm.

Electron microscopic examination of Sf101 phage preparation revealed that it has an isometric head (ca. 50 nm) and a short tail (ca. 16 nm) (Figure 1). According to the morphological classification of Bradley, these characteristic features are typical of phage in the C group morphology of the family *Podoviridae* and order *Caudovirale* [33]. Among the other serotype converting phages of *Shigella*, appearance of Sf101 phage resembles phage Sf6 and SfX [34, 35].

### Host range

Twelve serotypes (1a, 1b, 2a, 2b, 3a, 3b, 4a, 4b, 5a, 7a, X, and Y) of *S. flexneri* were tested for sensitivity against Sf101 phage. Together, Sf101 was able to infect 4 serotypes: 1a, 1b, 3b and Y. Interestingly, we observed some differences in the phage ability to form plaques on different serotypes. Sf101 plating efficiency was similar on serotypes Y and 1a; however, it was 10^5^ fold lower when plated on serotypes 3b and 1b. In a number of short-tailed phages, adsorption is mediated by an initial reversible binding of the phage to bacterial LPS, followed by an irreversible attachment of the phage to an unknown secondary receptor, the presence of which has been found to be necessary for infection. Recently, OmpA and OmpC were identified as the secondary receptors for bacteriophage Sf6, and were shown to dramatically affect the rate and efficiency of bacteriophage Sf6 infections [36]. Bacteriophage Sf101 being similar to Sf6 might also require presence of secondary receptors for infection, and the difference in the plating efficiencies observed might be due to mutation in the secondary receptors of these strains.

### Genome properties and organisation

The complete genome of Sf101 phage was sequenced using 314 chips on an Ion torrent PGM, producing an average read length of approx. 125 bp. A total of approx. 21,069 reads were generated which were then de-novo assembled into 6 major contigs using CLC Genomics Workbench (Ver 5.5.1, CLC Bio). The accuracy of the contigs was verified by mapping the reads back to the contigs. The regions of ambiguity and the gaps between the contigs were then addressed by targeted Sanger sequencing to obtain a single contiguous sequence.

The complete genome of Sf101 phage consists of 38,742 bp, with the overall G + C content of 47.4%. A total of 66 protein coding genes with a plausible Shine-Dalgarno sequence were predicted from the genome sequence. Among which 41 *orfs* are transcribed from the sense strand and 25 are on the antisense strand. The majority of the *orfs* initiate translation with an ATG, whereas only 6 start with GTG. The sizes of the gene products varied from 24 a.a. to 911 a.a.

**Figure 2:**
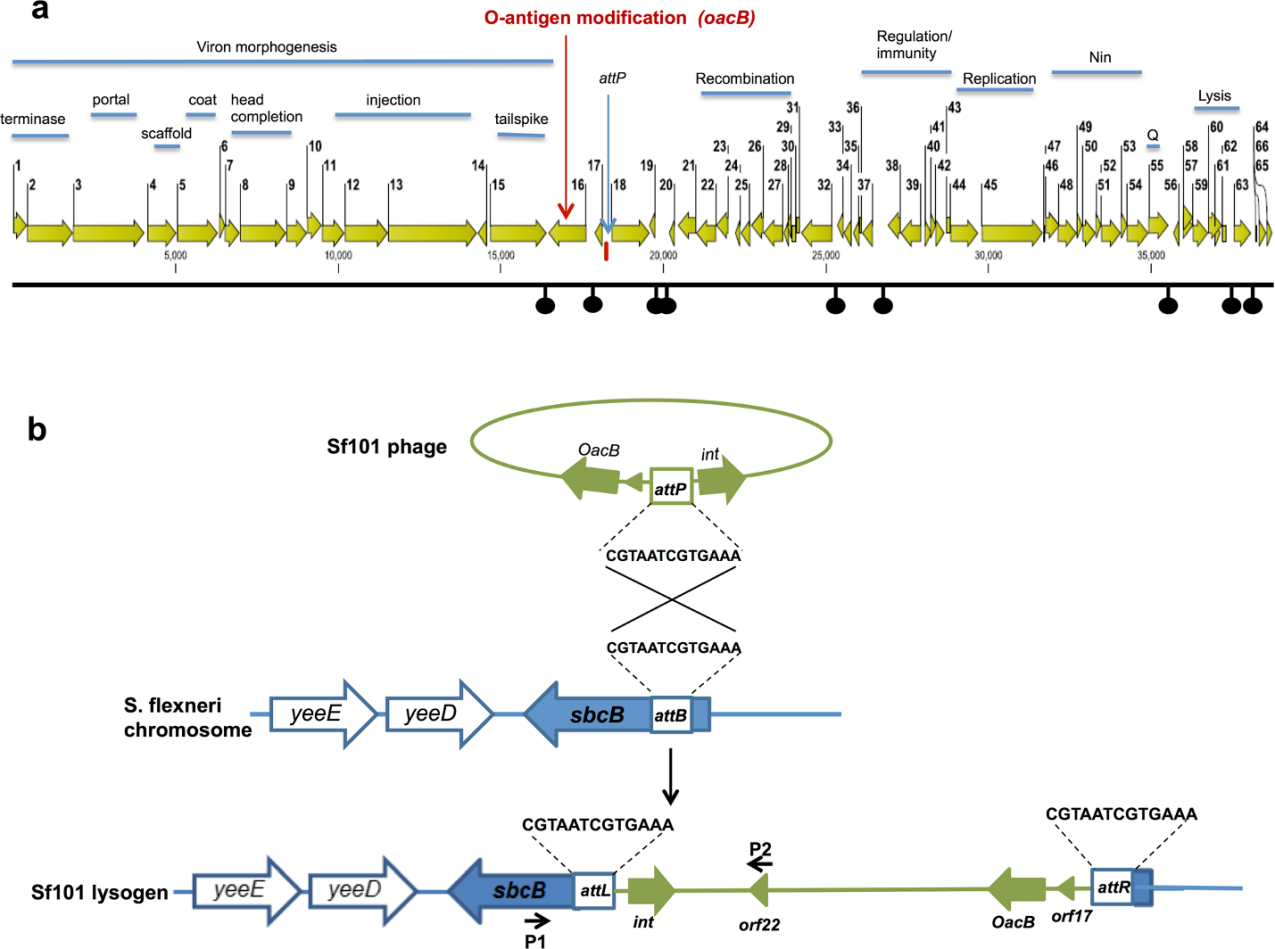
**Genome map and integration site of phage Sf101. (a)** The Sf101 genome is shown with a scale in bp. *orfs* are represented by arrows oriented in the direction of transcription. Putative functional modules are indicated above the arrows. Black knobs below the scale depict the Rho-independent terminators and the attachment site of the phage (*attP*) is indicated with a blue vertical arrow. **(b)** Schematic representation of phage Sf101 integrative recombination where *attP* on the phage genome recombines with the *attB* within *sbcB* gene of *S. flexneri* chromosome to form the two junction’s recombination sites *attL* and *attR*. Arrows P1 (sbcB-dwn-att-Fwd) and P2 (Orf22-up-Rev) on the map indicate the primers used in the PCR.

The translated *orf* products were also compared with known protein sequences using BlastP (Additional file 2: Table S2). Based on the similarities, 43 out of 66 *orfs* were assigned putative functions, while the other 23 *orfs* exhibited similarity to uncharacterised proteins. The overall genetic organisation of Sf101 phage (Figure 2a), consisting of "DNA packaging and structure - serotype conversion - regulation – recombination - replication – nin and the lysis region", suggested that this phage is a member of lambdoid family. Additionally, the layout of the genes was similar to that of previously sequenced serotype converting *Shigella* phages Sf6 and SfX.

The Sf101 genes were generally tightly spaced occupying 94.5% of the genome. However, there were several large (~200 to 600 bp) apparently non-coding regions within the genome (e.g. between *orfs* 16-17, 17-18, 19-20, 23-24 and 37-38). Although no tRNA genes were identified in these or other locations by using a tRNA scanning program, several putative Rho-independent transcription terminators were identified and are shown in Figure 2a.

### Relationship to other phages

**Figure 3:**
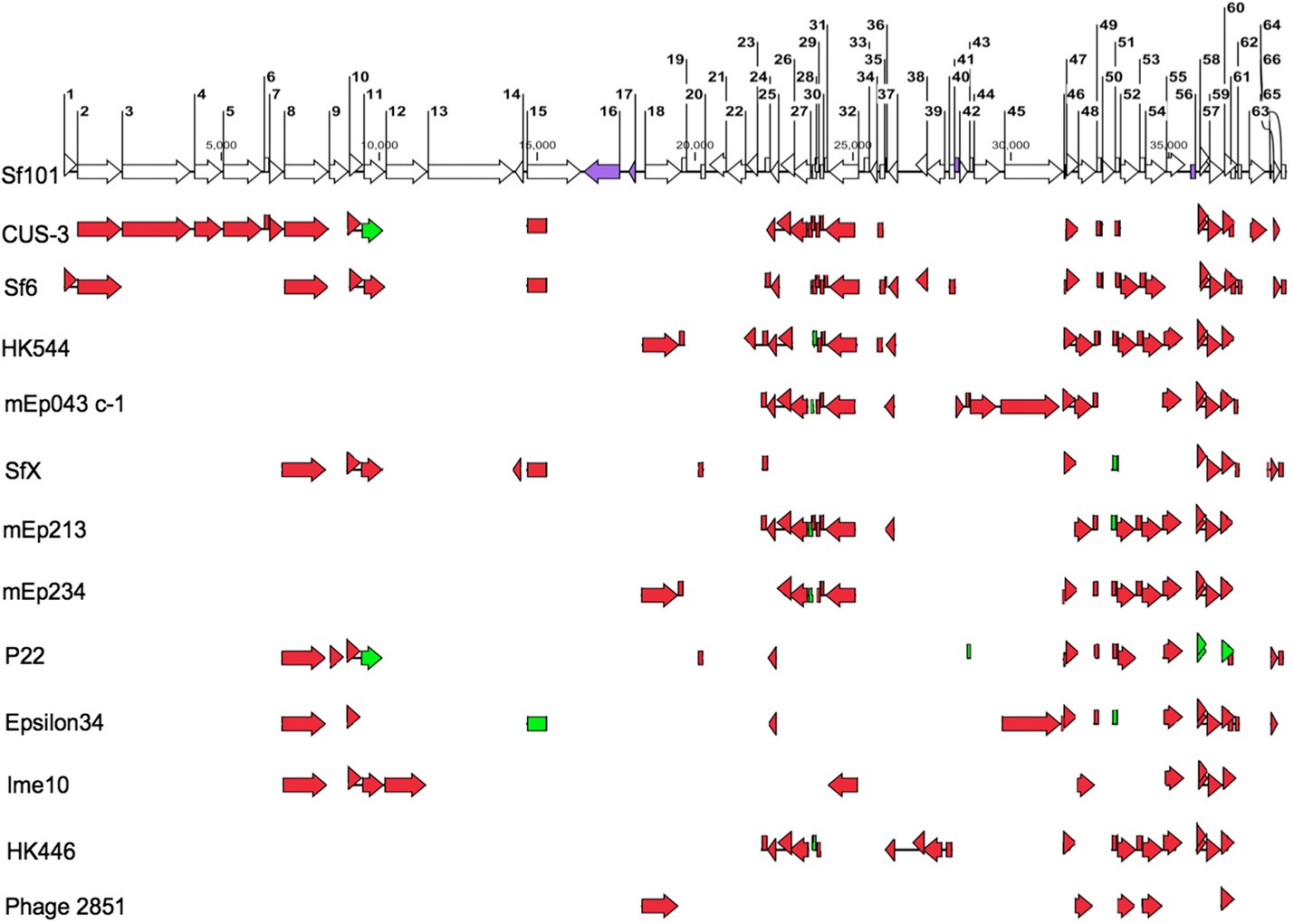
**Comparison of Sf101 with related phages.** Proteins encoded by Sf101 phage were compared with those of 12 other lambdoid phages from *E. coli, S. flexneri* and *Salmonella*. The coloured arrows represent the level of amino acid sequence identity between Sf101 protein and its counterpart in other phages. The red and green colours indicate >90% and >80% identity, respectively. The purple arrows on Sf101 map represent proteins with no phage-borne homologue.

Initial whole genome Blast of phage Sf101 against the NCBI database showed that bacteriophage Sf101 is related to several lambdoid phages originating from various hosts, like *S. flexneri*, *E. coli* and *Salmonella*. To better understand the relatedness, comparison of proteins encoded by Sf101 phage to those of the 12 most related phages was carried out. Figure 3 shows that most of the proteins encoded by the phage Sf101 were >90% identical with proteins from previously characterised lambdoid phages. Moreover, the genome of phage Sf101 is highly mosaic with the left half of the phage most homologous to *E. coli* phage CUS-3 and the right half most homologous to phage Sf6 and HK544.

The module containing DNA packaging and structural proteins is the largest module of the Sf101 phage, and is predicted to contain 15 *orfs* (*orf 1* to *15*). While the packaging proteins of Sf101 were identical to Sf6, most of the proteins responsible for phage head assembly shared >90% identity with their counterparts in CUS-3 (Figure 3). One of the exceptions was ORF9 in the head completion module. *orf9* with two other genes of the module encode for proteins required for stabilizing the phage head after DNA gets loaded in it. Although, ORF9 shared only 50% identity with its equivalent in CUS-3, it was found to be >90% identical to its homologue in phage P22. The major difference between CUS-3 and Sf101 head morphogenesis proteins, was in the DNA injection module. Sf101 ORF11, 12 and 13, forming this module showed only 85%, 34% and 32% identity to their equivalents in CUS-3. In general, the injection proteins are known to be more variable than the rest of the virion assembly proteins because these proteins are released by the virion during DNA injection and function in the host during or after the injection [37].

*orf15* of Sf101 encodes the tail spike protein that adsorbs to the cell receptors (O-antigen) of the host. Analysis of the tail spike protein of Sf101 phage revealed conservation in the amino terminal portion than in the carboxyl terminal region. The amino terminal residues were homologous to the cognate amino acid residues of CUS-3, Sf6, SfX, and epsilon34 (90, 90, 90, and 85% identity, respectively). However, very limited homology was observed between the carboxyl terminal residues. The reason for this could be that the amino terminal domain of the P22 like tail spikes are known to bind to virion head and is therefore more conserved. The carboxy terminal domain, on the other hand, contains the O-antigen binding site and so is specific for each phage [37, 38]. The integrase of phage Sf101 (encoded by *orf18*) shared 99% identity with the integrases of HK544, mEp234 and phage 2851. Moreover, further analysis of Sf101 genome sequence has revealed a 13 bp sequence (5’CGTAATCGTGAAA 3’) located upstream of the *int* gene, that is, identical to the *attP* of phage 2851 [39]. While the integration sites and *attP* sequence of HK544 and mEp234 are not known, phage 2851 has been shown to integrate in the *sbcB* locus [39]. The extremely high similarity of the Sf101 phage *attP* and Int protein to those of phage 2851 suggested that the integration site of Sf101 phage must be at the *sbcB* locus, identical to phage 2851. We amplified and sequenced one of the Sf101 prophage-bacterial genomic junctions that spans between Sf101 *orf22* and *sbcB* gene (Figure 2b). A 3 kb sequence was obtained in which 13 bp *attL* sequence was identified; thus confirming the integration of Sf101 phage into the 5’ end of *sbcB* gene (encoding 3’-5’ exonuclease). This is unlike all the other known serotype-converting phages of *S. flexneri* which are known to integrate either into the *agrW* or *thrW* tRNA gene [10, 15–18].

The early and regulatory regions located in the right half of the genome shared homology to CUS-3, Sf6, HK544, mEp043c-1, mEp213, mEp234 and HK446. However, the proteins of regulation and the replication module were most similar (>90% identical) to their cognates in HK446 and mEp043c-1, respectively. The nin region of the Sf101 phage (ORF46-54) was identical to that of phage HK544, with ninX being the only exception showing only 43% identity. Moreover, nin E, F, G, H, and I of Sf101 phage were also conserved in Sf6, mEp213, mEp234 and HK446 phages.

The antitermination Q and a set of predicted lysis genes lie downstream of the nin region. The lysis module of phage Sf101 is typical of lambdoid phages, consisting of holin, anti-holin, lysin, Rz and Rz1 proteins (encoded by *orfs 57-61*). Additionally, *orf61* of Sf101 phage lies inside *orf60*, in a second reading frame, as in other lamdoid phages [38]. Homologues of holin, anti-holin, lysin, and Rz were seen in all the phages shown in Figure 3, except for phage P22 and phage 2851. In contrast, equivalents of Rz1 of phage Sf101 were only present in CUS-3, Sf6, P22 and epsilon34. To the right of the lytic cassette, *orfs 62, 64, 65* and *66* encoded proteins which shared >90% identity with their homologues in SfX and Sf6, whereas *orf63* encoded Rha family protein was identical to its cognate in phage CUS-3.

### Novel genes of Sf101 phage

Sf101 phage proteins encoded by *orfs 16, 17, 41* and *56*, were identified as proteins with no known phage-borne homologues. Whilst ORF17 and 56 shared similarity with uncharacterised proteins of *E. coli*, ORF41 had no homologue in *E. coli* or *Shigella* and showed limited homology with proteins from *Saprospira grandis* and *Bipolaris maydis*. BlastP results of ORF16 revealed that it is an acyltransferase family protein, sharing 99% identity with the protein encoded by gene SF0315 (named *oacB*) of *S. flexneri* 2a str 301. OacB has recently been shown to confer the 3/4-O-acetylation modification of Rha^III^ of *S. flexneri* O-antigen [23].

Pairwise alignments of Sf101 *orf16* (Sf101-*oacB*) with the *oacB* at the DNA and protein levels were carried out. The analysis identified three base substitutions (G486 to A, C692 to T and G796 to A) in the Sf101-*oacB*. While the first mutation had no effect at the protein level, the other two mutations resulted in two amino acid substitutions in Sf101-OacB: A231 to V and V266 to I (Additional file 3: Figure S1). Since, A, I and V are all neutral amino acids with similar structure, it was expected that these mutations will impose minimum effect on the function of OacB. This was verified by performing NMR spectroscopy experiments.

### Confirming the function of Sf101-OacB

In order to confirm the function of Sf101-OacB, we first analysed the LPS of the Sf101 host (SFL1683) by ^1^H NMR spectroscopy. Results revealed NMR resonances at *δ*_H_ 2.21 and 2.17 indicating the presence of O-acetyl groups [40, 41]. *oacB* gene from Sf101 phage was then cloned into a plasmid (pNV2074) and introduced into a serotype 7a strain (SFL1691), which was PCR negative for *oacB* gene, to create a recombinant strain SFL2516. The ^1^H NMR spectrum acquired on the LPS of strain SFL2516 revealed resonances, inter alia, at *δ*_H_ 2.21, 2.17 and 2.15; however, NMR signals were absent in the spectral region for O-acetyl groups for the SFL1691 LPS.

**Figure 4:**
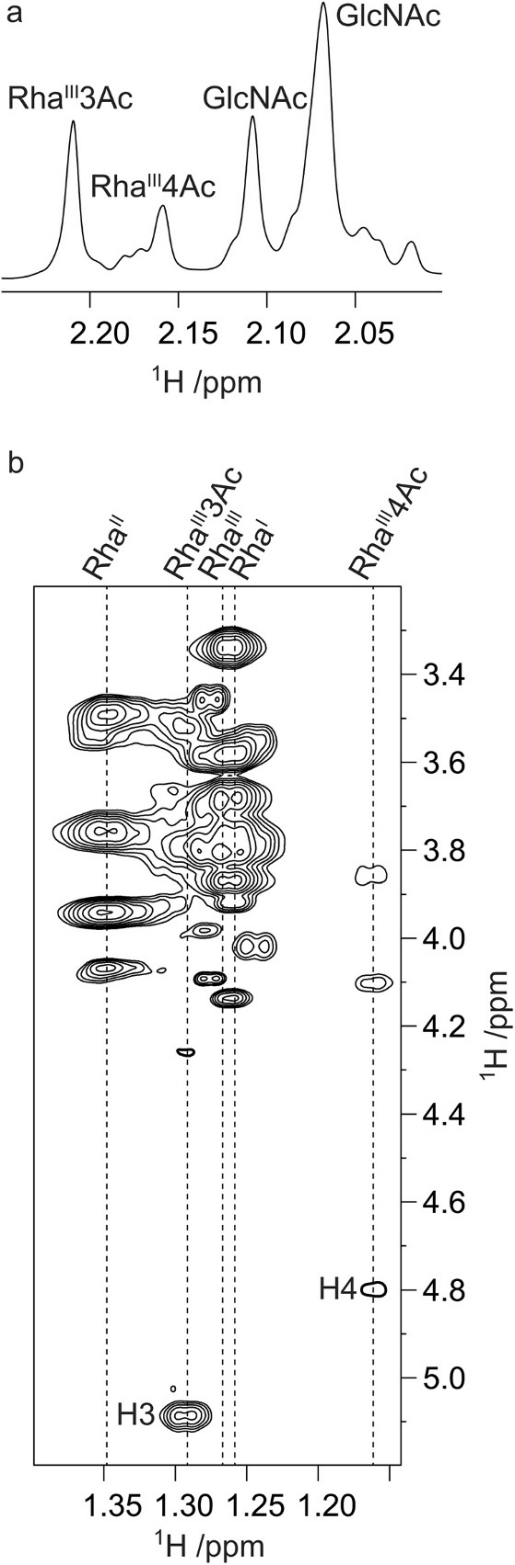
**NMR.** Selected regions of **(a)** ^1^H NMR spectrum of the PS from strain SFL1683 showing resonances from O- and N-acetyl groups and **(b)** ^1^H,^1^H-TOCSY (τ_mix_ 120 ms) NMR spectrum of the PS from strain SFL2516 showing spin systems originating from the H6 protons in the methyl groups of rhamnosyl residues. Annotations are given with respect to the O-acetylation pattern in the O-antigen.

LPS of strains SFL1683 and SFL2516 were then treated by dilute aqueous acetic acid and purified by size exclusion chromatography to obtain the polysaccharide materials (PS). The ^1^H NMR spectra of these PS showed resonances, inter alia, at *δ*_H_ 2.21 (0.8 H), 2.16 (0.5 H), 2.11 (0.9 H) and 2.07 (2.1 H) for the SFL1683 strain (Figure 4a) and at *δ*_H_ 2.22 (0.8 H), 2.17 (0.7 H), 2.13 (0.8 H) and 2.08 (2.2 H) for the SFL2516 strain, indicating partial O-acetyl substitution. The O-acetyl substitution position(s) were then determined by performing the ^1^H,^1^H-TOCSY experiments employing an array of mixing times of increasing lengths, which have proven to be particularly powerful at unraveling the spin-systems of the sugar residues and subsequently the O-acetyl substitution position(s) [42]. Using mixing times in the range 10–120 ms, the ^1^H,^1^H-TOCSY spectra of the PS from strain SFL2516 revealed, inter alia, spin systems originating from *δ*_H_ 1.29 (H6 of Rha^III^3Ac) to 3.79 (H5), 3.53 (H4), 5.09 (H3) and 4.26 (H2), from *δ*_H_ 1.16 (H6 of Rha^III^4Ac) to 3.86 (H5), 4.80 (H4), 4.10 (H3) and 4.22 (H2), as well as from *δ*_H_ 1.26 (H6 of Rha^III^) to 3.68 (H5), 3.34 (H4), 3.87 (H3) and 4.14 (H2) (Figure 4b). The ^1^H,^1^H-TOCSY spectra of the PS from strain SFL1683 were closely similar, and taken together these results were fully consistent with the NMR data reported by Wang et al. [23] for partial O-acetyl substitution at positions O3 and O4 of Rha^III^ in O-antigens from other *S. flexneri* serotypes. Thus, O-acetylation was present to about 1/4 at O3 and to about 1/5 at O4 in the two strains investigated herein.

### Identification of conserved residues and motifs of OacB

BlastP results of Sf101-OacB also revealed that this protein shared some homology with acyltransferases from other species. All the homologues were grouped under the superfamily COG1835 (acyl_transf_3). Multiple alignment of the Sf101-OacB with 20 of its homologues from various species was carried out for identification of the conserved residues and motifs. Results of alignment (Additional file 4: Figure S2) showed several conserved motifs: DGxRGxLAxxVxxHH, FFxITG(YorF)LFxxK, WxLxYEWxFY and YSXYLxHG (where x is any amino acid), present at similar positions of the proteins. The first 3 motifs were found in the amino terminal part of the protein while the fourth one was in the carboxy terminus. Several other conserved amino acids were also identified in close proximity to these motifs. The high levels of conservation of various amino acid motifs suggests that they may be involved in specific conserved functions.

### Distribution of *oacB*in *S. flexneri*7a strains

The 3/4-O-acetyl modification observed in this study was in a serotype 7a strain. However, serotype 7a is known to lack any O-acetyl modification to date [21]. Thus, several serotype 7a isolates from different regions (4 from Bangladesh, 9 from Egypt, 1 from Sweden, 31 from UK, and 14 from Vietnam) were screened to identify a possible 3/4-O-acetylation modification. As the antiserum specific for 3/4-O-acetyl modification was not available, a PCR screening of 59 serotype 7a strains with *oacB* specific primers was performed. As shown in Table 1 the expected PCR product was amplified from 7 out of 59 strains (1 isolate each from Sweden, UK, and Egypt, and 4 from Bangladesh). The sequencing of the PCR product from the positive strains revealed that the *oacB* gene sequence in 2 strains (1 from Egypt and 1 from Sweden) was identical to *oacB* of Sf101 phage (Additional file 3: Figure S1). However, the *oacB* gene in the other 5 strains was almost identical to *oacB* from *S. flexneri* 2a str 301 except a base substitution at position 1146, which resulted in I382 to M mutation in the OacB protein of all 5 strains. However, as I and M are neutral amino acids, this mutation is expected to have no effect on the function of OacB. These results suggest that the 3/4-O-acetyl modification in serotype 7a is not uncommon. Moreover, in order to determine if *oacB* gene in these 7a strains was carried by Sf101 phage, PCRs targeting three different regions of Sf101 phage (*orf15*-*orf16*, *orf30*-*orf39*, and *orf60*-*orf66*) were performed on the above 7 *oacB* positive strains. Results revealed that 1 out of the 7 tested strains carried complete Sf101 phage suggesting this phage may have contributed in the dissemination of the *oacB* gene in 7a strains (Table 1).

**Table 1:** **PCR screening of** ***oacB*** **in serotype 7a isolates from various regions**

Serotype 7a isolates obtained from	Number of strains screened	Number of ***oacB***PCR positive strains	Number of Sf101 phage PCR positive strains	Location of ***oacB***
**Bangladesh**	4	4	0	Upstream of *adrA*
**Egypt**	9	1	1	*sbcB*
**Sweden**	1	1	0	Upstream of *adrA*
**UK**	31	1	0	Upstream of *adrA*
**Vietnam**	14	0	-	-

Integration site of Sf101 phage suggests that the *oacB* gene in Sf101 lysogens is located in the *sbcB* locus. However, the reported site for *oacB* gene in serotype 1a, 1b, 2a, 5a and Y is upstream of the *adrA* gene (which encodes diguanylate cyclase AdrA) [23]. Thus, the location of *oacB* gene in the 7 *oacB* positive serotype 7a strains was determined by performing PCR using primer specific for *oacB* gene and *adrA*. Results indicate that the location of *oacB* in the Sf101 lysogen was the *sbcB* locus, while in the other 6 strains *oacB* was located upstream of the *adrA* gene. Change in the chromosomal position of *oacB* from *sbcB* to *adrA* locus is likely due to disruption of the prophage by IS elements and mobilization of *oacB* due to the combination of 2 or more IS elements flanking *oacB*. This hypothesis is supported by our analysis of the region flanking *sbcB* gene in 10 previously sequenced *S. flexneri* genomes obtained from the NCBI database. Results revealed that the region upstream of *sbcB* gene is extremely variable containing insertion elements in all, and Sf101 like Int in 8 out of 10 strains. Taken together, our analysis indicate that a possible presence and disruption of Sf101 phage by IS elements could have resulted in the mobilization of *oacB* in some and loss in the other strains. Additionally, Wang et al. have also reported that the *oacB* gene located upstream of *adrA* in serotypes 1a, 1b, 2a, 5a & Y was carried by a transposon-like structure [23].

## Conclusion

This study reports the isolation and characterisation of a novel bacteriophage Sf101 from *S. flexneri* serotype 7a strain. Complete genome sequence of Sf101 phage was determined and found to contain functional *oacB* gene making it a serotype converting phage of *S. flexneri*. The comparative genomic analysis of Sf101 to 12 other lambdoid phages revealed that bacteriophage Sf101 has a highly mosaic genome and Sf6-like genome organisation. Sf101 was found to integrate in the *sbcB* locus representing a new genomic location of *oacB* gene in Sf101 lysogen. Additionally, this study for the first time identified *oacB* gene in several serotype 7a isolates from different regions, providing evidence of 3/4-O-acetylation modification in serotype 7a of *S. flexneri.* These findings will further our understanding on the serotype converting phages of *Shigella* which will be useful in comprehending the role these bacteriophages play in the survival of *S. flexneri* in the environment and human host.

## Electronic supplementary material

Additional file 1: Table S1: List of primes used in this study. (DOCX 17 KB)

Additional file 2: Table S2: Analysis of predicted *orfs* and proteins of Sf101. (DOCX 26 KB)

Additional file 3: Figure S1: Multiple alignment of the amino acid sequence of OacB from Sf101, *S. flexneri* 2a str 301, and 7 serotype 7a strains performed using ClustalW. Amino acid substitutions in Sf101 OacB when compared with OacB of *S. flexneri* 2a str 301 are boxed in green. Residues highlighted in red are identical to OacB from Sf101, while the ones in green share identity with OacB of *S. flexneri* 2a str 301. Residues in grey are point mutations in the protein sequences of serotype 7a strains. (DOCX 21 KB)

Additional file 4: Figure S2: Alignment of Sf101 OacB with homologues acyltansferases. The OacB protein from Sf101 phage was aligned with its homologues in several other species using ClustalW. Regions highlighted in pink are conserved amino acids. Conserved motifs are shown by green lines above the alignment. (DOCX 42 KB)
