# Supplementary material for: Serotype-conversion in Shigella flexneri: identification of a novel bacteriophage, Sf101, from a serotype 7a strain
Source: BMC Genomics. 2014 Aug 30;15(1):742. doi: 10.1186/1471-2164-15-742 (PMC4159516; doi:10.1186/1471-2164-15-742)
Supplement: Supplementary file 1 — Additional file 1: Table S1: List of primes used in this study. (DOCX 17 KB) [file 12864_2014_6412_MOESM1_ESM.docx]

Table S1: List of primers used in this study

| Primer name | Primer Sequence | Target site |
| --- | --- | --- |
| sbcB-dwn-att-Fwd | ATGACATTGAATTCGTCATCGGT | *sbcB* gene |
| sbcB-revcom-Rev | CCTACCTTCCTCTTTCACG | *sbcB* gene |
| Orf22-up-Rev | TACTGAATGCCATCCATCCATTT | Sf101 *orf22* |
| Dwn-Sf101oacB-Fwd | AGATAGTCAATGGAATTGCC | Sf101 *oacB(orf16)* |
| AdrA-Rev | TTTTGAGGAGTTAAAGGAGGTTC | *adrA* gene |
| Sf10I-oacB-Fwd | AGCGAGCTCTGCAAAGCTTGGTGTGTCT | Upstream of Sf101 *oacB* gene |
| Sf101-oacB-Rev | TGCTCTAGAGAATCTCCAGTTCGTTTGC | Sf101 *oacB* |
| EmR-PvuI-Fwd | AATCGATCGTAAGACGGTTCGTGTTCGT | Upstream erythromycin gene |
| EmR-PvuI-Rev | AATCGATCGCATAGAATTATTTCCTCCCG | Downstream erythromycin gene |
| Tail-down-fwd | TTCGTGCCCATTACGAAGG | Sf101 *orf15* |
| Dwn-kil-Fwd | TTGATGGCTATTTCATTGCG | Sf101 *orf30* |
| Up-CI-Rev | CGTGGAAGCGAGGGTAAA | Sf101 *orf39* |
| Dwn-Rz-Fwd | AAGTGGAATCGACAGGATAA | Sf101 *orf60* |
| Sf6-g68-up-Rev | GCTCGTATAGCTCTTGAGT | Sf101 *orf68* |
