# Supplementary material for: Serotype-conversion in Shigella flexneri: identification of a novel bacteriophage, Sf101, from a serotype 7a strain
Source: BMC Genomics. 2014 Aug 30;15(1):742. doi: 10.1186/1471-2164-15-742 (PMC4159516; doi:10.1186/1471-2164-15-742)
Supplement: Supplementary file 2 — Additional file 2: Table S2: Analysis of predicted orfs and proteins of Sf101. (DOCX 26 KB) [file 12864_2014_6412_MOESM2_ESM.docx]

Table S2: Analysis of predicted *orfs* and proteins of Sf101.

| ***orf*** | **Product** | **Region** | **Size (a.a)** | **Related products (sizes)** | **Accession no.** | **BlastP evalue (% identity)** |
| --- | --- | --- | --- | --- | --- | --- |
| 1 | Phage terminase, small subunit | 7..429 | 140 | gene 1 protein [140aa, Enterobacteria phage Sf6], terminase small subunit [140aa, Salmonella phage HK620] | NP_112075.1 | 1E-97 (100) |
| 2 | Phage terminase, large subunit | 426..1841 | 471 | phage terminase, large subunit, pbsx family [471aa, Escherichia coli HS], PBSX family phage terminase, large subunit [471aa, Escherichia coli KTE28] | YP_001457081.1 | 0.0 (100) |
| 3 | Portal protein | 1843..4041 | 732 | portal protein [732aa, Escherichia coli KTE96]; portal protein [732aa, Escherichia coli UMEA 3592-1] | WP_000818366.1, WP_021572772.1 | 0.0 (99), 0.0 (99) |
| 4 | Scaffold protein | 4132..5025 | 297 | scaffold protein [297aa, Escherichia sp. TW09308], putative aTPase involved in DNA repair [297aa, Escherichia coli 174750] | WP_000372585.1; WP_004015027.1 | 0.0 (99), 0.0 (99) |
| 5 | Coat protein | 5044..6297 | 417 | hypothetical protein ECCG_00842 [417aa, Escherichia coli B088]; P22 coat - protein 5 family protein [417aa, Escherichia coli 174750] | WP_000013272.1, WP_004015029.1 | 0.0 (100), 0.0 (99) |
| 6 | Unknown | 6339..6527 | 62 | hypothetical protein ESRG_00800 [62aa, Escherichia coli TA124]; hypothetical protein ECRS218_0017 [62aa, Enterobacteria phage CUS-3] | WP_001389518.1, YP_008572345.1 | 1E-37 (100), 2e-37 (98) |
| 7 | Phage DNA stabilization protein | 6508..6969 | 153 | putative head completion protein [153aa, Enterobacteria phage CUS-3]; DNA stabilization protein from phage [153aa, Escherichia coli S88] | YP_541646.1 | 4E-106 (99) |
| 8 | DNA stabilization protein | 6979..8397 | 472 | DNA stabilization protein [472aa, Escherichia coli KTE6], hypothetical protein HMPREF1606_01489 [472aa, Escherichia coli 908522 | WP_001576812.1, WP_023145936.1 | 0.0 (99), 0.0 (99) |
| 9 | DNA stabilization protein | 8401..9039 | 212 | putative DNA stabilization protein [212aa, Salmonella enterica subsp. enterica serovar Montevideo str. 4441 H], tail needle protein gp26 [212aa, Escherichia coli KTE234] | WP_000774918.1, WP_001549445.1 | 3E-148 (99) |
| 10 | Head assembly protein | 9039..9494 | 151 | head assembly protein [151aa, Escherichia coli HVH 146 (4-3189767)]; head assembly protein [151aa, Escherichia coli UMNF18] | WP_000627631.1, WP_000627634.1 | 1E-108 (100), 2e-108 (99) |
| 11 | DNA transfer protein | 9497..10189 | 230 | putative DNA transfer protein [230aa, Shigella flexneri 2002017]; DNA transfer protein [230aa, Enterobacteria phage IME10] | YP_687809.1 | 4E-154 (99) |
| 12 | DNA injection protein | 10199..11530 | 443 | prophage DNA injection protein [443aa, Shigella flexneri 2a str. 301]; putative prophage DNA injection protein [443aa, Shigella flexneri 2002017] | NP_706180.1 | 0.0 (98) |
| 13 | Transglycosylase | 11531..14266 | 911 | transglycosylase SLT domain-containing protein [911aa, Escherichia coli E1520], injection domain protein [657aa, Shigella flexneri VA-6] | WP_000035073.1, WP_005066135.1 | 0.0 (99), 0.0 (99) |
| 14 | putative Chromosome segregation ATPase | complement(14284..14577) | 97 | putative Chromosome segregation ATPase [97aa, Shigella flexneri 2002017], Chromosome segregation ATPase [99aa, Escherichia coli P12b] | YP_005725983.1, YP_006168161.1 | 4e-61 (97), 5e-57 (92) |
| 15 | Bifunctional tail protein | 14666..16408 | 580 | bifunctional tail [570aa, Shigella flexneri VA-6], head binding family protein [446aa, Shigella flexneri K-404] | WP_001279737.1, WP_005119065.1 | 0.0 (86), 0.0 (77) |
| 16 | O-acyltransferase B | complement(16462..17634) | 390 | hypothetical protein SF0315 [390aa, Shigella flexneri 2a str. 301], acyltransferase family protein [390aa, Shigella flexneri K-218] | NP_706267.1 | 0.0 (99) |
| 17 | Unknown | complement(17881..18129) | 82 | hypothetical protein WC9_02130 [153aa, Escherichia coli KTE231], hypothetical protein ECRM13514_2658 [48aa, Escherichia coli O145:H28 str. RM13514] | WP_016231943.1, AHG09331.1 | 4e-17 (62), 0.39 (62) |
| 18 | Integrase | 18400..19578 | 392 | Mobile element protein [392aa, Escherichia coli IS5], integrase [398aa, Escherichia fergusonii ATCC 35469] | CDK52401.1, YP_002383214.1 | 0.0 (99), 0.0 (99) |
| 19 | Unknown | complement(19559..19750) | 63 | hypothetical protein HK140_030 [63aa, Enterobacteria phage HK140], transcriptional regulator AlpA family [63aa, Enterobacteria phage HK544] | YP_002271252.1 | 2e-39 (100) |
| 20 | Eag protein | complement(20167..20346) | 59 | phage EaG protein [Escherichia coli AA86], eag family protein [Escherichia coli P0299438.10] | WP_001277762.1 | 3E-34 (100) |
| 21 | Unknown | complement(20443..21009) | 188 | hypothetical protein G970_02398 [239aa, Escherichia coli UMEA 3341-1], hypothetical protein G899_02444 [239aa, Escherichia coli UMEA 3022-1] | WP_021571723.1, WP_021552870.1 | 7E-113 (72), 1e-111 (71) |
| 22 | Unknown | complement(21006..21623) | 205 | hypothetical protein WCM_02673 [245aa, Escherichia coli KTE10], hypothetical protein G723_01880 [245aa, Escherichia coli HVH 50 (4-2593475)] | WP_001515048.1, WP_023277044.1 | 5e-71 (96), 3e-70 (96) |
| 23 | Unknown | complement(21620..22000) | 126 | conserved hypothetical protein [180aa, Escherichia coli B185], hypothetical protein ECDEC9E_3592 [123aa, Escherichia coli DEC9E] | WP_001307745.1, WP_001430997.1 | 9E-74 (93), 9E-71 (90) |
| 24 | Unknown | complement(22200..22364) | 54 | hypothetical protein HK620p09 [54aa, Enterobacteria phage HK620], hypothetical protein SF660363_0185 [54aa, Shigella flexneri 6603-63] | NP_112042.1 | 2e-30 (100) |
| 25 | Anti-RecBCD protein | complement(22375..22671) | 98 | Anti-RecBCD protein 2 [98aa, Escherichia coli 541-15], Anti-RecBCD protein 2 [98aa, Escherichia coli CUMT8] | WP_001111280.1 | 1e-65(99) |
| 26 | Unknown | complement(22695..23078) | 127 | phage protein [127aa, Escherichia coli UMEA 3199-1], hypothetical protein mEp213_038 [127aa, Enterobacterial phage mEp213] | WP_021563808.1, YP_006134682.1 | 2e-88 (100), 2e-87 (99) |
| 27 | Recombination protein | complement(23078..23683) | 201 | DNA single-strand annealing protein; essential recombination function protein Erf [211aa, Escherichia coli S88], recombination protein [201aa, Enterobacteria phage CUS-3] | YP_002392222.1, YP_541683.1 | 1E-145 (99), 2e-145 (99) |
| 28 | Unknown | complement(23694..23864) | 56 | gene 29 protein [56aa, Enterobacteria phage Sf6], hypothetical protein HK106_038 [56aa, Enterobacteria phage HK106] | NP_958205.1 | 1E-33 (98) |
| 29 | Unknown | complement(23785..23943) | 52 | gp42.1 [52aa, Escherichia coli DEC8D], gene 30 protein [52aa, Enterobacteria phage Sf6] | WP_000010964.1, NP_958206.1 | 9e-30 (100), 4e-29 (98) |
| 30 | Kil protein | complement(23940..24092) | 50 | kil protein [50aa, Enterobacteria phage IME10], gene 31 protein [50aa, Enterobacteria phage Sf6] | WP_016248932.1, NP_037729.1 | 4e-27 (100), 9e-27 (98) |
| 31 | CIII protein | complement(24077..24208) | 43 | gene 32 protein [43aa, Shigella phage Sf6], CIII protein [43aa, Enterobacteria phage HK633] | NP_958208.1 | 8e-22 (100) |
| 32 | Unknown | complement(24233..25201) | 322 | hypothetical protein WCG_04391 [322aa, Escherichia coli KTE6], hypothetical protein ECDEC12D_2685 [322aa, Escherichia coli DEC12D] | WP_001576834.1, WP_000005782.1 | 0.0 (99), 0.0 (99) |
| 33 | Unknown | complement(25336..25539) | 67 | hypothetical protein ERCG_02269 [66aa, Escherichia coli E1520], hypothetical protein HMPREF9540_03653 [70aa, Escherichia coli MS 115-1] | WP_000333101.1, WP_000968444.1 | 2E-38 (100), 2e-16(63) |
| 34 | Unknown | complement(25536..25787) | 83 | hypothetical protein G723_01891 [83aa, Escherichia coli HVH 50 (4-2593475)] , hypothetical protein [45aa, Escherichia coli IS25] | WP_023277052.1, CDK85392.1 | 9e-52 (95), 3e-21 (96) |
| 35 | Restriction inhibitor protein | complement(25829..26029) | 66 | restriction inhibitor protein ral [66aa, Escherichia coli KTE210], lambda ant-restriction protein [66aa, Escherichia coli UTI89] | WP_001542700.1, NP_753451.1 | 4e-41 (98), 1e-40 (98) |
| 36 | Unknown | complement(26013..26099) | 28 | hypothetical protein lambdap47 [28aa, Enterobacteria phage lambda], hypothetical protein SfI_0043 [28aa, Enterobacteria phage SfI] | NP_040623.1 | 3E-27 (100) |
| 37 | Anti-termination protein N | complement(26108..26434) | 108 | anti-termination protein N [108aa, Enterobacteria phage HK633], antitermination protein [108aa, Enterobacteria phage HK544] | YP_007112320.1 | 9e-72 (100) |
| 38 | Unknown | complement(26895..27299) | 134 | hypothetical protein G750_02478 [134aa, Escherichia coli HVH 88 (4-5854636)], gene 38 protein [134aa, Enterobacteria phage Sf6] | WP_021526961.1, NP_958214.1 | 1e-91 (99), 4e-90 (98) |
| 39 | Repressor cI | complement(27296..27928) | 210 | prophage repressor [210aa, Enterobacteria phage HK446], prophage repressor CI [210aa, Shigella flexneri 5 str. 8401] | YP_007111993.1, YP_687851.1 | 1e-151 (100), 3e-151 (99) |
| 40 | Cro | 28032..28247 | 71 | gene 40 protein [71aa, Enterobacteria phage Sf6], prophage antirepressor [71aa, Enterobacteria phage HK446] | NP_958215.1 | 3e-43 (100) |
| 41 | Unknown | 28198..28383 | 61 | glycosyl transferase family 2 [264aa, Saprospira grandis], hypothetical protein COCHEDRAFT_1199156 [288aa, Bipolaris maydis C5] | YP_005324443.1, EMD85023.1 | 2.0 (34), 4.0 (37) |
| 42 | CII protein | 28364..28645 | 93 | CII protein [93aa, Enterobacteria phage mEp043 c-1], transcriptional activator protein C1 [93aa, Shigella flexneri K-404] | YP_007111552.1 | 2e-60 (100) |
| 43 | Unknown | 28680..28826 | 48 | hypothetical protein Stx1_gp57 [56aa, Stx1 converting phage], hypothetical protein HK620p23 [48aa, Enterobacteria phage HK620] | YP_003848880.1, NP_112056.1 | 6E-26 (100), 8e-26 (100) |
| 44 | Replication protein O | 28819..29679 | 286 | gp59 [286aa, Escherichia coli B7A], phage replication protein O domain [286aa, Escherichia coli HVH 175 (4-3405184)] | WP_000067062.1, WP_021537883.1 | 0.0 (100), 0.0 (99) |
| 45 | Unknown | 29787..31667 | 626 | hypothetical protein mEp043_055 [626aa, Enterobacteria phage mEp043 c-1], putative replication protein [626aa, Escherichia coli CUMT8] | YP_007111555.1 | 0.0 (100) |
| 46 | NinA | 31668..31748 | 26 | gp63 [26aa, Enterobacteria phage ES18], ninA protein [26aa, Enterobacteria phage P22] | YP_224201.1, YP_063725.1 | 4e-20 (100), 4e-19 (100) |
| 47 | NinB | 31745..32185 | 146 | recombination protein NinB [146aa, Enterobacteria phage SfI] , NinB protein [146aa, Enterobacteria phage HK446] | NP_753463.1 | 5e-102 (100) |
| 48 | DNA methylase | 32122..32709 | 195 | phage N-6-adenine-methyltransferase [195aa, Escherichia coli H736], phage N-6-adenine-methyltransferase [195aa, Escherichia coli M605] | YP_002412152.1, WP_001311519.1 | 3E-140 (99) |
| 49 | NinE protein | 32706..32882 | 58 | NinE protein [58aa, Enterobacteria phage HK630], NinE protein [Enterobacteria phage HK629] | YP_002555863.1, YP_007111828.1 | 4e-34 (100), 5e-34 (100) |
| 50 | NinX protein | 32885..33295 | 136 | hypothetical protein A193_01398 [136aa, Escherichia coli KTE234], Putative protein ninX [138aa, Escherichia coli] | WP_001549090.1, WP_000177652.1 | 4e-97 (100), 9e-97 (99) |
| 51 | NinF protein | 33288..33464 | 58 | protein ninF [69aa, Escherichia coli KTE102], NinF [58aa, Enterobacteria phage ST104] | WP_016244746.1, YP_006390.1 | 1e-33 (98), 6E-33 (98) |
| 52 | NinG protein | 33457..34068 | 203 | protein ninG [203aa, Escherichia coli UMEA 3329-1], bacteriophage Lambda NinG protein [204aa, Escherichia coli E22] | WP_021571394.1, WP_001299176.1 | 3e-145 (99), 3e-145 (99) |
| 53 | NinH protein | 34065..34271 | 68 | gene 57 protein [68aa, Enterobacteria phage Sf6], NinH protein [68aa, Enterobacteria phage HK630] | NP_040640.1 | 2E-42 (100) |
| 54 | NinI protein | 34249..34914 | 221 | serine/threonine-protein phosphatase [223aa, Escherichia coli HVH 223 (4-2976528)], NinI protein [221aa, Enterobacteria phage lambda] | WP_021546261.1, NP_040641.1 | 1E-161 (99), 2e-160 (99) |
| 55 | Antitermination Q | 34911..35534 | 207 | antitermination protein Q [207aa, Escherichia coli B088], late gene regulator Q [207aa, Enterobacteria phage HK106] | WP_001235462.1, YP_687858.1 | 2e-153 (100), 1e-152 (99) |
| 56 | Unknown | complement(35675..35866) | 63 | hypothetical protein HMPREF1606_00980 [63aa, Escherichia coli 908522], hypothetical protein [63aa, Escherichia coli IS5] | WP_023145899.1, CDK52213.1 | 8E-34 (92), 2e-33 (92) |
| 57 | Holin | 35968..36291 | 107 | gene 60 protein [107aa, Enterobacteria phage Sf6], holin [107aa, Enterobacteria phage HK544] | NP_958234.1 | 8E-70 (100) |
| 58 | Anti-holin | 35974..36291 | 105 | gene 61 protein [105aa, Shigella phage Sf6], holin-like protein [105aa, Salmonella enterica subsp. enterica serovar Paratyphi C strain RKS4594] | NP_958235.1 | 3E-68 (100) |
| 59 | Lysozyme | 36275..36751 | 158 | lysozyme [158aa, Enterobacteria phage HK620], lysin [158aa, Enterobacteria phage mEpX2] | NP_037753.1 | 3e-113 (100) |
| 60 | Rz (i-spanin) | 36748..37185 | 145 | phage endopeptidase [145aa, Escherichia coli KTE28], enterobacteria phage Sf6 gene 63 protein [145aa, Escherichia coli LF82] | WP_001527418.1, YP_002555873.1 | 5e-98 (99), 1e-97 (99) |
| 61 | Rz1 (o-spanin) | 36947..37132 | 61 | outer membrane lipoprotein Rz1 [61aa, Escherichia coli IHE3034], lipoprotein precursor [61aa, Enterobacteria phage CUS-3] | YP_006101799.1 | 7E-34 (97) |
| 62 | Unknown | 37173..37325 | 50 | hypothetical protein SFxv_0335a [50aa, Shigella flexneri 2002017], hypothetical protein ECMT8_14924 [50aa, Escherichia coli CUMT8] | YP_005725966.1 | 3e-26 (100) |
| 63 | Rha family protein | 37532..38074 | 180 | Phage Rha protein [180aa, Escherichia coli CE516], phage regulatory protein Rha [180aa, Escherichia coli H252] | YP_008572352.1 | 7E-131 (100) |
| 64 | Unknown | 38187..38261 | 24 | hypothetical protein SFxv_0335c [24aa, Shigella flexneri 2002017] | YP_005725968.1 | 5e-21 (100) |
| 65 | Unknown | 38302..38544 | 80 | hypothetical protein HK620p40 [80aa, Enterobacteria phage HK620], hypothetical protein Sf6p67 [80aa, Enterobacteria phage Sf6] | NP_112073.1 | 8e-50 (100) |
| 66 | Unknown | 38546..38725 | 59 | hypothetical protein ECEG_01700 [59aa,Escherichia coli B354], hypothetical protein HK620p41 [59aa, Enterobacteria phage HK620] | YP_002383170.1, NP_112074.1 | 2E-32 (98), 7e-32 (97) |
