# Supplementary material for: Serotype-conversion in Shigella flexneri: identification of a novel bacteriophage, Sf101, from a serotype 7a strain
Source: BMC Genomics. 2014 Aug 30;15(1):742. doi: 10.1186/1471-2164-15-742 (PMC4159516; doi:10.1186/1471-2164-15-742)
Supplement: Supplementary file 3 — Additional file 3: Figure S1: Multiple alignment of the amino acid sequence of OacB from Sf101, S. flexneri 2a str 301, and 7 serotype 7a strains performed using ClustalW. Amino acid substitutions in Sf101 OacB when compared with OacB of S. flexneri 2a str 301 are boxed in green. Residues highlighted in red are identical to OacB from Sf101, while the ones in green share identity with OacB of S. flexneri 2a str 301. Residues in grey are point mutations in the protein sequences of serotype 7a strains. (DOCX 21 KB) [file 12864_2014_6412_MOESM3_ESM.docx]

**Figure S1**

Sf101-OacB MHMIEINSLLLITSVILMSLLAVGLFDKISPINLVEHGRNNQIDGMRGFLAIFVLIHHAAIWNGYLSSGVWEAPSSNLLANLGQVGVSFFFMITGYLFFSKIISGDQDWTRLYVSRLLRL 120

Sflex2a-str301-OacB MHMIEINSLLLITSVILMSLLAVGLFDKISPINLVEHGRNNQIDGMRGFLAIFVLIHHAAIWNGYLSSGVWEAPSSNLLANLGQVGVSFFFMITGYLFFSKIISGDQDWTRLYVSRLLRL 120

1-Bangladesh-str7a MHMIEINSLLLITSVILMSLLAVGLFDKISPINLVEHGRNNQIDGMRGFLAIFVLIHHAAIWNGYLSSGVWEAPSSNLLANLGQVGVSFFFMITGYLFFSKIISGDQDWTRLYVSRLLRL 120

2-Bangladesh-str7a MHMIEINSLLLITSVILMSLLAVGLFDKISPINLVEHGRNNQIDGMRGFLAIFVLIHHAAIWNGYLSSGVWEAPSSNLLANLGQVGVSFFFMITGYLFFSKIISGDQDWTRLYVSRLLRL 120

3-Bangladesh-str7a MHMIEINSLLLITSVILMSLLAVGLFDKISPINLVEHGRNNQIDGMRGFLAIFVLIHHAAIWNGYLSSGVWEAPSSNLLANLGQVGVSFFFMITGYLFFSKIISGDQDWTRLYVSRLLRL 120

4-Bangladesh-str7a MHMIEINSLLLITSVILMSLLAVGLFDKISPINLVEHGRNNQIDGMRGFLAIFVLIHHAAIWNGYLSSGVWEAPSSNLLANLGQVGVSFFFMITGYLFFSKIISGDQDWTRLYVSRLLRL 120

Sweden-str7a MHMIEINSLLLITSVILMSLLAVGLFDKISPINLVEHGRNNQIDGMRGFLAIFVLIHHAAIWNGYLSSGVWEAPSSNLLANLGQVGVSFFFMITGYLFFSKIISGDQDWTRLYVSRLLRL 120

Egypt-str7a MHMIEINSLLLITSVILMSLLAVGLFDKISPINLVEHGRNNQIDGMRGFLAIFVLIHHAAIWNGYLSSGVWEAPSSNLLANLGQVGVSFFFMITGYLFFSKIISGDQDWTRLYVSRLLRL 120

UK-str7a MHMIEINSLLLITSVILMSLLAVGLFDKISPINLVEHGRNNQIDGMRGFLAIFVLIHHAAIWNGYLSSGVWEAPSSNLLANLGQVGVSFFFMITGYLFFSKIISGDQDWTRLYVSRLLRL 120

Sf101-OacB TPMFIVSLCLIFIIVGFKSGWRMQVSTEELFVSIMKWLPFTALGMPNINDVKDSFTINAAVTWTLVYEWFFYFSLPVISALIKRKVSIYMVMISAISLFVFILFFSKIHIVSFLFGLLAF 240

Sflex2a-str301-OacB TPMFIVSLCLIFIIVGFKSGWRMQVSTEELFVSIMKWLPFTALGMPNINDVKDSFTINAAVTWTLVYEWFFYFSLPVISALIKRKVSIYMVMISAISLFVFILFFSKIHIASFLFGLLAF 240

1-Bangladesh-str7a TPMFIVSLCLIFIIVGFKSGWRMQVSTEELFVSIMKWLPFTALGMPNINDVKDSFTINAAVTWTLVYEWFFYFSLPVISALIKRKVSIYMVMISAISLFVFILFFSKIHIASFLFGLLAF 240

2-Bangladesh-str7a TPMFIVSLCLIFIIVGFKSGWRMQVSTEELFVSIMKWLPFTALGMPNINDVKDSFTINAAVTWTLVYEWFFYFSLPVISALIKRKVSIYMVMISAISLFVFILFFSKIHIASFLFGLLAF 240

3-Bangladesh-str7a TPMFIVSLCLIFIIVGFKSGWRMQVSTEELFVSIMKWLPFTALGMPNINDVKDSFTINAAVTWTLVYEWFFYFSLPVISALIKRKVSIYMVMISAISLFVFILFFSKIHIASFLFGLLAF 240

4-Bangladesh-str7a TPMFIVSLCLIFIIVGFKSGWRMQVSTEELFVSIMKWLPFTALGMPNINDVKDSFTINAAVTWTLVYEWFFYFSLPVISALIKRKVSIYMVMISAISLFVFILFFSKIHIASFLFGLLAF 240

Sweden-str7a TPMFIVSLCLIFIIVGFKSGWRMQVSTEELFVSIMKWLPFTALGMPNINDVKDSFTINAAVTWTLVYEWFFYFSLPVISALIKRKVSIYMVMISAISLFVFILFFSKIHIVSFLFGLLAF 240

Egypt-str7a TPMFIVSLCLIFIIVGFKSGWRMQVSTEELFVSIMKWLPFTALGMPNINDVKDSFTINAAVTWTLVYEWFFYFSLPVISALIKRKVSIYMVMISAISLFVFILFFSKIHIVSFLFGLLAF 240

UK-str7a TPMFIVSLCLIFIIVGFKSGWRMQVSTEELFVSIMKWLPFTALGMPNINDVKDSFTINAAVTWTLVYEWFFYFSLPVISALIKRKVSIYMVMISAISLFVFILFFSKIHIASFLFGLLAF 240

Sf101-OacB LLNKSKIVNGIAKAKVTPIIITAIMIFEMTYFKTTYAPLPLILCGITFIIIASGCDLYGILRLNITRKLGETTYSVYLLHGIFLYCLMTWIIPNNYTENTFIILVSTTAFLITFTSCLTF 360

Sflex2a-str301-OacB LLNKSKIVNGIAKAKVTPIIITAIMVFEMTYFKTTYAPLPLILCGITFIIIASGCDLYGILRLNITRKLGETTYSVYLLHGIFLYCLMTWIIPNNYTENTFIILVSTTAFLITFTSCLTF 360

1-Bangladesh-str7a LLNKSKIVNGIAKAKVTPIIITAIMVFEMTYFKTTYAPLPLILCGITFIIIASGCDLYGILRLNITRKLGETTYSVYLLHGIFLYCLMTWIIPNNYTENTFIILVSTTAFLITFTSCLTF 360

2-Bangladesh-str7a LLNKSKIVNGIAKAKVTPIIITAIMVFEMTYFKTTYAPLPLILCGITFIIIASGCDLYGILRLNITRKLGETTYSVYLLHGIFLYCLMTWIIPNNYTENTFIILVSTTAFLITFTSCLTF 360

3-Bangladesh-str7a LLNKSKIVNGIAKAKVTPIIITAIMVFEMTYFKTTYAPLPLILCGITFIIIASGCDLYGILRLNITRKLGETTYSVYLLHGIFLYCLMTWIIPNNYTENTFIILVSTTAFLITFTSCLTF 360

4-Bangladesh-str7a LLNKSKIVNGIAKAKVTPIIITAIMVFEMTYFKTTYAPLPLILCGITFIIIASGCDLYGILRLNITRKLGETTYSVYLLHGIFLYCLMTWIIPNNYTENTFIILVSTTAFLITFTSCLTF 360

Sweden-str7a LLNKSKIVNGIAKAKVTPIIITAIMIFEMTYFKTTYAPLPLILCGITFIIIASGCDLYGILRLNITRKLGETTYSVYLLHGIFLYCLMTWIIPNNYTENTFIILVSTTAFLITFTSCLTF 360

Egypt-str7a LLNKSKIVNGIAKAKVTPIIITAIMIFEMTYFKTTYAPLPLILCGITFIIIASGCDLYGILRLNITRKLGETTYSVYLLHGIFLYCLMTWIIPNNYTENTFIILVSTTAFLITFTSCLTF 360

UK-str7a LLNKSKIVNGIAKAKVTPIIITAIMVFEMTYFKTTYAPLPLILCGITFIIIASGCDLYGILRLNITRKLGETTYSVYLLHGIFLYCLMTWIIPNNYTENTFIILVSTTAFLITFTSCLTF 360

Sf101-OacB KLIETPFIKLTKQTTTLVKELIPTLTNNNQ 390

Sflex2a-str301-OacB KLIETPFIKLTKQTTTLVKELIPTLTNNNQ 390

1-Bangladesh-str7a KLIETPFIKLTKQTTTLVKELMPTLTNNNQ 390

2-Bangladesh-str7a KLIETPFIKLTKQTTTLVKELMPTLTNNNQ 390

3-Bangladesh-str7a KLIETPFIKLTKQTTTLVKELMPTLTNNNQ 390

4-Bangladesh-str7a KLIETPFIKLTKQTTTLVKELMPTLTNNNQ 390

Sweden-str7a KLIETPFIKLTKQTTTLVKELIPTLTNNNQ 390

Egypt-str7a KLIETPFIKLTKQTTTLVKELIPTLTNNNQ 390

UK-str7a KLIETPFIKLTKQTTTLVKELMPTLTNNNQ 390
