# Supplementary material for: Serotype-conversion in Shigella flexneri: identification of a novel bacteriophage, Sf101, from a serotype 7a strain
Source: BMC Genomics. 2014 Aug 30;15(1):742. doi: 10.1186/1471-2164-15-742 (PMC4159516; doi:10.1186/1471-2164-15-742)
Supplement: Supplementary file 4 — Additional file 4: Figure S2: Alignment of Sf101 OacB with homologues acyltansferases. The OacB protein from Sf101 phage was aligned with its homologues in several other species using ClustalW. Regions highlighted in pink are conserved amino acids. Conserved motifs are shown by green lines above the alignment. (DOCX 42 KB) [file 12864_2014_6412_MOESM4_ESM.docx]

**Figure S2**

Sf101 phage 14 SVILMSLLAVGLFDKISPINLVEHGRNNQIDGMRGFLAIFVLIHHAAIWNGYLSSGVWEAPSSNLLANLGQVGVSFFFMITGYLFFSKIISGDQ-----D 108

Rhizobium sp. CF080 24 ACMMFASGLAVKSALISPVVTASSNRYGALDGLRGLLAYGVMFHHSMAAYSYFSSGVWYGLDNAVVFQLGKTTVALFFMITGFLFTEKCITGKVN----- 118

Thiothrix nivea 22 VFMFIVASYFIRQDTFAKSLTVPSGRYGSLDGLRGVLATGVFIHHAFAMYVYFSGGEWQWSTSPVFNQLGQTTVSLFFMITGFLFTLKALAPTID----- 116

Cupriavidus metallidurans CH34 15 LCLALVWPKR-LWRFLDDPPSGQGQRYVTVDGLRGFLALAVVLHHCVISYGFHQTGEWKLPPSSFYSIIGQVGVSIFFMITAFLFWGRLLDQGKR---LD 110

Burkholderia sp. CCGE1003 14 AILCCLFALP-IFAFVDD-AAGVKERMQTIDGLRGFLAASVMVYHGLINYNYVTVGKWMAADALFYRPLGGVAVMFFFMITAFLFWTRLLRRDGR---PD 108

Azospirillum sp. B510 14 VAVCVSWAILRLIAPRLRPQPGSDARYAGIDGLRGLLAFAVFIHHGVITWQYLHTGVWALPPSRLHTHLGQSGVALFFMVTAFLFWDKLLKAGPG---MD 110

Pseudomonas aeruginosa 11 VCVALALLTCGIIRYLKP-IPLPLSRFSTIDGLRGYLAFFVFLHHAAIWYYFLRSGAWQVPPSNLYTHFGQTSVSLFFMITGFLFTHKLLQSKNR--PID 107

Nitrospira defluvii 51 AALLLALGTATLLPEITPRQATSPVRYTSLDGLRGYLAFAVFLSHSSIWYFYLRSGTWDVPPSNFYAHLGQSSVILFFMITAFLFWSKLLDGRVQ--PVD 148

Competibacter phosphatis 11 LILYVAVVVAYLMSRYSE-IPPDQGRFVALDGLRGYLALFVFLYHSALWYFYLRTGQWEVPPSNLYTHFGQSSVLLFFMITGFLFFSKLLDGRTR--GID 107

Dechloromonas aromatic RCB 11 FAILMALFSCFVLIRKFG-PPEEQGRYLAIDGLRGYLAFCVFLHHSIIWFFYLKTNQWAVPPSNLYTHFGQTGVAFFFMITGFLFFSKILEGRGK--EID 107

Pseudomonas sp. GM55 11 VAVAFALLSTLAISSQLP-PPSSHGRFLTIDGLRGYLAFFVFLHHSCIWYYYLQTGAWVAPPLRLFVHFGQVGVSLFFMITGFLFFSKIMDDRGR--GVD 107

Pseudomonas fluorescens F113 11 IAILVALLSTNLFLGKLV-VPSSHGRFVTLDGLRGYLAFFVFLHHSYIWYYYIHSDVWVLPSSRLFVHFGQVGVALFFMITGFLFFNKLLEGRAR--GID 107

Rhodobacter capsulatus 11 IVFSIALITVFLLAGLLR-IVPQDDRVSTIDGLRGYLACGVFLHHSAIWYYYLHTGKWVAPPSHLYAHLGQTSVALFFMITGFLFYSKILSSR----PLD 105

Pseudomonas sp. S9 8 IATGIALTAALCVQKIVP-VHDSSHRHTAIDGMRGYLAFFVFLHHSSIWYFYLHEGRWALPPSNLFSHLGQSSVALFFMITGFLFYSKILSSRDK--KIS 104

Flavobacterium columnareATCC49512 11 IIFFIAFTTAYIINLKFK-IINNNTRYETIDGIRGFLAIGVFIHHASIWFQYLQIKSWVAPKSNLYNQLGQTSVSLFFMITSFLFITKLLNSENQ--KIN 107

Klebsiella pneumonia MGH78578 4 KYISNKLGIELINS----------GRFTAIDGLRAYLSLFVFFHHFFIFYKWKESGKWEKPEILFIDDLGQVSVAIFFIITGFLFIGKITSGR----HFD 89

Serratia fonticola 14 AMIFCVLFMKIINSRLIKIELPIGNRAGCIDGARGYLALMVAVHHYYIFYFWIKTGKWLPPEVQYINSLGKVAVSAFFIITGYLFLGKILRDNRLHIKTQ 113

Acinetobacter pittii 18 SVTIFSLIFTKIN----ILPETNSGRTSTIDGLRGILALSVMTHHFYITYIWKTVGEWKKPENILIDNFGGVAVSLFFLITGYLFISKIRKDE-----VS 108

Enhydrobacter aerosaccus 14 SLTLVTWLLNQLPQRM-IVGFDASHRHSPLDGIRGILALSVFTHHFFKNYFFQTTGRWQSPNIDFFTNLGSVPVSLFFLITGYLFFGKLKQPW-----MD 107

Burkholderia ambifaria 28 VAIIAWLVARHVPFYRSAVDKSLSMRHPNLDGFRGILATSVVFHHFACNQGLLSTGVWGAP-SDFLRNLGTIPVALFFMVTGFLFWERACRGT-----LD 121

Sf101 phage WTRLYVSRLLRLTPMFIVSLCLIFIIVGFKSGWRMQVSTEELFVSIMKWLPFTAL-GMPNINDVKDSFTINAAVTWTLVYEWFFYFS-LPVISALIKRKV 206

Rhizobium sp. CF080 WRSLYISRLFRLAPLYILVMLVMFTVIFALSGFQVLVPISKLGSQLGKWLLFGAV-GRPNINGFEKSWTLIAGVNWSLRFEWQFYLFGLPVLFLMSRFMN 217

Thiothrix nivea WKRLYASRVLRLVPLYALIVSSIFGLVFYLSNWALNEPVWEIIKEYWVWMTFVCF-GRPDINAYPMSWTLIANVNWSLKYEIMFYLFAVPAIHVFSRFVQ 215

Cupriavidus metallidurans CH34 WFALYVNRFLRIAPLYWVVVALMLLVVAIKTGFTLAVPPSELVKQVFQWALPAIVRGMPPVNGYQQTSTITAGVTWTLYYEWMFYFS-LPFLAIAAIKRS 209

Burkholderia sp. CCGE1003 WAALYIGRFFRIAPLYIFAVLAMTFIVFWRSGFELREQLGIFLPNMGRWLAFGLNDDMQPINAFPWTIFILMGVTWSIKYEWWFYFS-LVVSAVFVRTGM 207

Azospirillum sp. B510 WTGFLSSRFHRLYPVYAVAVLLTMILALAATGFEFRTGPLDLLRRLIGWATFKAP----PINGLENAGQIVAYATWSLPYELLFYAA-LPALALLVSPVR 205

Pseudomonas aeruginosa WLQLYISRFMRIYPAYIFAIAIMFTIAFFMTGYTLHESVLSLLKKTIQWGAFRTP----DINGLVETRRIMAGVTWTLPYEWLFYLC-LPALSLLIGRRA 202

Nitrospira defluvii WSRLYLARVFRLVPLYLLVVIGVVSIALYRAGFRLQESMTSFALHLASWLTFTIP-GIAPVNGFAET-IPLAGAVWSLPYEWLFYAC-LPIGSWCIRAAT 245

Competibacter phosphatis WLKLFIGRLLRLVPLYLFMIVSLCIIVMVVSGWVLHEPLLKIAEHLVCWLGFTIL-GAPDLNGVKDTLLIVSGVIWTLPYEWFFYLI-LPVLAMTVRVVP 205

Dechloromonas aromatic RCB WLRLYVSRFLRLMPLYAVAMGALFLIVFSLSGATLHEPISVLASEVLRWMAFTVR-GNPDINGIERTSLIMAGVTWSLPYEWCFYML-LPVFALVNRVRV 205

Pseudomonas sp. GM55 WLRLYVSRFLRLTPLYFFSMILLFSIVGWVTKENQTDSFSELIGACLKWLGFTIYFGVPDINGFPGTYVVNAGVTWTLPYEWTFYMA-LPLIALAIGVWP 206

Pseudomonas fluorescens F113 WFRLYVSRFLRLTPLYLFSMVLLFLIVWVLTKNEPAQPTGKLIVDGLKWIGFRVS-GAPDLNGLLGTRYIHAGVTWTLPYEWFFYLF-LPFVALAIGSRP 205

Rhodobacter capsulatus WTRIFVSRLLRLTPLYLVVISLMLIVVGVQTQWELREPLYNLINNIIRWFLFLQA----NVNQMEETRTITAGVTWTLPYEWSFYFL-LPALALFTGR-- 198

Pseudomonas sp. S9 WTYIYTSRFMRLVPLYLLAISILLLFVFIKTNWKIETGLSSILIDATKWVLFTIP-GAPDINNVKHTSLIMAGVTWSLPYEWLFYFS-LPLIALTTGLSR 202

Flavobacterium columnare ATCC49512 WPIIFISRFFRLVPMYLVSIFLLISIVFIISDWQLNVTPFKLLKEVLQWGTFTIL-SSPTINDLSFTHIINAGVVWSLPYEWLFYFS-LPIISILIFKKK 205

Klebsiella pneumoniae MGH78578 FFSVYISRFFRIMPLYLLVVMITLFYSFLLTGLSIQIPHSELLKDIVRWLLFVGD----SVNGYTDAKRITAGVTWTLKYEWLFYFS-LPLLFLILKNKV 184

Serratia fonticola WSPLYLSRFLRITPLYLLSVLVTIILTFVLSEGVLYVSFFELFKEVSRWLLYVGY----SINGDADARRITAGVTWTLKYEWVFYLT-LPILAILLKERT 208

Acinetobacter pittii WKQIYISRIKRIIPLYLFVFLFILAITLLN-VQITASNYIEFLKWVSDWILFKGG----SFQ-NFESGLVIAGVHWTLIYEWKFYFA-LPLIFVIWQ-QK 200

Enhydrobacter aerosaccus WQYLYRSRLQRIVPLYLFLGICIVAIYFT--QQHPPLPLSEWQQWTLRWLSFNNQ----SLK-DFYAWPLVAGAAWTLLYEWGFYFS-LPLLFVLVHPKQ 199

Burkholderia ambifaria VRSFFVGRVRRVAPLYLFYATVIVACTLYWFPRNVVDAPIRLLADLLKAVSLGWF-GAFPVNGAEHTGYLSG-VWWTLAYEWRFYVA-IPFLAWFVATRA 218

Sf101 phage SIYMVMISAISLFVFILFFS--------KIHIVSFLFGLLAFLLNKSKIVNGIAKAKVTPIIITAIMIFEMTYFKTTYA-PLPLILCGITFIIIASGCDL 297

Rhizobium sp. CF080 RKALLTGVLTGLGTLALYGWIRGDLGLELLCTAHFLCGIAASLIYNNSVGKRLITSRAFHAAALFCLPVLATSPQGTVV--PDLLATFAIFLSVLGGGSL 315

Thiothrix nivea LRAALVLCAAMLGAILLFRWQRGGEGGISLYTAQFLGGVMVAYAFKIDRLKAVIEGNIIRWLAAGALVSLFFMQYAYSA--VAVIGNIILFAAVVGGASL 313

Cupriavidus metallidurans CH34 PLAFVPACIWIVFVMPETLSS----AFARNLVAMFVMGMVAASLVRRSPGFRGD-SVLKSAIAVALLAFPLLTRSTAYE-SVSILSLGAFFILVSSGASL 303

Burkholderia sp. CCGE1003 HMVFALVGLAASFAIILTTQS----DLWC-LPAAFFCGMVTASLLHENVRPRML-ENTMSLIALAALALLFSFAKTHAG-VVQIALTGIAFYMMACGADL 300

Azospirillum sp. B510 RLRPALVSLVLTLALLLYFR-----NFSLNVLQCFLGGIAAAYAIRQPRFVRFARSDRGLVLALAALLATVAGFPGAYA-PGPVLGLGLFFAIVAAGQDF 299

Pseudomonas aeruginosa PMT-ALATTIIIASLILSFW-----RPSPILLCMFLAGGIAALTARSEWLQSLSNGRLGSLLCVCLIGSAVIIFPTAYT-LGPAILLSLAFILITAGCSI 295

Nitrospira defluvii PTSWLLFSAAAVVTLTTVMP-----DVRSPVLYAFGGGIAAASLARVDAIRTRLTHRVRGLVVVACLGITMWLFETAYT-VPALLLLTGAFTIIACGNSI 339

Competibacter phosphatis PPLYIVLSVGSIVTFIIYNPQTHPVHPQLHPLFSFFGGIAASLLVRSDSFRWFCRKDYCSFLVIGLIIAVVVLFQTAYA-IMPLIFISLIFSLIAGGNSL 304

Dechloromonas aromatic RCB GRVYIIFSV-LVLIFIFKN------HTYMYHWLSFVGGIAAAYLVRIDYLRYLLRKKVFSMAAILLISATVAFFPSTYD-WAPIAMLSAVFVMIAAGNGV 297

Pseudomonas sp. GM55 PVKYLLLSVLSMYAFGYLY------LAGSFYWL-FLGGMVAAILVRFESFRAVAVSRWATLIVLVLAVYVVTMYPSVYDYVQAKFLLVIVFCFIAGGNSI 299

Pseudomonas fluorescens F113 PVKYLCLAAIALYVFDVYG------YSWSFGWL-FLGGMVAAVLVRYDRFTIFSVSKWATCLIVGSLAWSVTYYPTIYESSVPRLLLIAVFCLISGGNTL 298

Rhodobacter capsulatus PVPVIILPIVALSAFYMYFAG-----LRPSRFVFFLGGIGAAFLARRSWFCQLAAHKASSLVILASMTCLITLFPSAYG-KIQLVLIFIAFSLVAAGNSL 292

Pseudomonas sp. S9 N-LTAAAVSLAVVLLIYTVFD-----PATIHIKTFIDGMLCAALHHMKIMTGLSSSRLYSAFIIILLYVLVTTYQSSHG-NIQILILSLVFFGISGGNNL 295

Flavobacterium columnare ATCC49512 TSFFYTVISLFFILCFFKIYG-----SSIPHLLSFLGGIIPPFIIKYNTKKINFNSNFYS-IIILLCLGLILLFHTSDN-YICKLLIIIVFNLIALGNEM 298

Klebsiella pneumonia MGH78578 ATTLIIIVCMLLLINNFKFHSI-IDSRYFIFFMIGGISNYICKLLENNIKLIEILRNRLISIILMVLLIYVFFSGVGIFNIFSIISLLLFFIAIVCGNNL 283

Serratia fonticola LAIISLIVGVG--ISYFNINGFGFESHYLIYFIFGGGIAFVQSL--NNKKLSGMMSTSIWSVISILSLVFVIFITPHYSVFLSDILILFFLMPIIFGNSL 304

Acinetobacter pittii IPKWIS-SILVIAF--MVYIFKHKSHHLYALFFL----AIPAVLYKDRFKQFMQTKPAITHIVLGILSIIVLFFTEAYS-WFQMLSLAVIFSFIVSGYS- 291

Enhydrobacter aerosaccus VCQWQNIAMLMISLPVLYYVFTHTIIKLYWLFLL----AFFAVWLEDFFKNLLAKFPVVFAIALCLLTLFILFETSAYS-YWQMLLCGLLFCFIANGFTY 294

Burkholderia ambifaria WRKLLALAIVTFCAALFGPPG--------GMALLFAFGALAFEASRHRAIRSLLGTKSAATVALLILAVAPYP-AERYS-IEGALPLLPVFLCVACGNTF 308

Sf101 phage YGILRLNITRKLGETTYSVYLLHGIFLYCLMTWIIPNNYTENTF-----IILVSTTAFLITFTSCLTFKLIETPFIKLTKQTTTLVKELIPTLTNNNQ-- 390

Rhizobium sp. CF080 WGLLKTRAAIWLGDISYGVYLIHGLALWLSYYNLSRAGLLDAID-GPIFLLLLPAIGLVVLAISSVSYIRVELPMIKLG--RKLGSPKTSSRVELA---- 408

Thiothrix nivea FGLLKHKSAVWLGDISYGIYLMHGLVLWLVLNMLAKAGILSQLG-LVGYWLVTFATAASIVLLASCSYALLEKPVMGLLPARKQDRVRGYKQPGLRTW-- 410

Cupriavidus metallidurans CH34 FGLLASRSAVRLGSVSYGIYLLQGIVITVLHSP-RVLGAFACKG-PEQFWLTTIAVGLVLVCVAAASYHFVERPCIRLGKKIGKAPESARQTHVEMGDGT 401

Burkholderia sp. CCGE1003 FGLLRLKSAQRLGHVSYGVYLLQGIPITLLFWH-APFRAWAVVS-PMHYWLALLVCAALLCVVAALTFALIERPFIQLGKSIHSPAS-------WLKAGA 391

Azospirillum sp. B510 RGTLTRQPVLWLGEISYGVYLLHGIVLWTLITANGPLRALIGAD-ESLYLLALTVAGVLVVALASLVHLTVERPAIRFGKRSRDPRQ------------A 386

Pseudomonas aeruginosa FGLLNLSVSRFFGEITYSLYLLHGIVLYVIFEIVLGNQAAKEFD-TRQHWMIIYAATPITVVLSYLSFRYIERPSMSASKGLGARIKS----LLRNTYRP 390

Nitrospira defluvii YGILEWPTSVLLSEMGYSLYLLHSLLLFAVYRLVLG-EWASTLS-ATEHWSVVLALVPILIVLCFATFRLIEQPAMAAVPRCHAWLVA----HLNE--RP 431

Competibacter phosphatis FGILRSSISRVLGELSCSIYLLHGMALFILFKFVVGPNHAQLLS-AQQHWMLVIAISPVILFISYWTFRLIEQPAMRATNTVTVWVSSRLTWRFRTYVVS 403

Dechloromonas aromatic RCB FGLLTNAVSRALGELAYSIYLLHGIVLFLLFRYVIGFEKSKLLQ-PIDYWSAIIAVSPILIFISYLSYRKIERPALLSTDGLTSWLRN----KINSYSEA 392

Pseudomonas sp. GM55 FGVLNSKVSRVMGEMAYSIYLLHGLVLFVAFRLVIGASLGKALS-PSQYWGVIVTLTPVLILICGLTFRFIERPAMKMTGPLTDWIRA----KKTNVIAG 394

Pseudomonas fluorescens F113 FGLLKLKVSLVMGEMAYSMYLLHGVLLFVIFRFIFGGARASELT-PLQYWGVIVLATPILMMICGLTFRFVERPAMRSVDTLTHWIRA----KKKGRFER 393

Rhodobacter capsulatus FGALTNRVSRALGEITYSIYLLHGLILYSLMKLVLFPDTNAALPSPFAFWCIVLCVTPVLITMSMLTFKFIEQPAMQHADTLATRLKASWTAFRSRDARA 392

Pseudomonas sp. S9 FGVLSWKTSRVLGEMAYSIYLLHGLILFTTFNFIINSETSIELT-PYQYWILIFAIAPAVVIISAITFVMIEKPSMDKTQKIANQICN---LSSKK---- 387

Flavobacterium columnare ATCC49512 FGVLKNTTLKFLGEISYSTYLIHGIIIFITLYFGFSLEVVEKMS-PSTFCSIIFLITPIIILTSFLSYRNIEKPFMDYSKKINYDKIN---YSITEFYKR 394

Klebsiella pneumoniae MGH78578 FGALTLKGARLLGEISYSIYLIHGCVIFSIF-ILMNKFSGLS-LSEYLILMPFVTIA--VVFISSLTYRFIEAPCINIGKKIQNIYRFNGKKAL------ 373

Serrati afonticola FGLLKLKSSIRLGEISYSIYLMHGIVFFVIF-IFLFDVNKMNGWVEFFIYMPFSLML--VVFFSVYTYQYIEKPFVDLSHIIR-KKRINTNEII------ 394

Acinetobacter pittii FGILNHKGLKVLGEISYSIYLIHGLVLYTIFTVINIVDLKTISLEKYYSFFLPTALL--VTIVSLFTYKFIECPFLRRPLKKL----------------- 372

Enhydrobacter aerosaccus FGVLYQQGLKVLGELSYSLYLTHGLVMYIWFNLLHLHDFS-QSMTAYVWTYPLVLVF--VVMFAVIGYNYIEKPLMHRPNSRVLSP-------------- 377

Burkholderia ambifaria YGVLQTRPLALLGTASFSIYMLHMAIVYALVRAFNKFVFPINSVDDSGIWALSFACALVAVFCALLTYRYIEHPFIAKRNDARPARRTIEPAREQSTAAL 408
